# The Two NF-κB Pathways Regulating Bacterial and WSSV Infection of Shrimp

**DOI:** 10.3389/fimmu.2019.01785

**Published:** 2019-07-30

**Authors:** Chaozheng Li, Sheng Wang, Jianguo He

**Affiliations:** ^1^State Key Laboratory for Biocontrol, School of Marine Sciences, Sun Yat-sen University, Guangzhou, China; ^2^Guangdong Provincial Key Laboratory of Marine Resources and Coastal Engineering, Sun Yat-sen University, Guangzhou, China; ^3^Southern Laboratory of Ocean Science and Engineering, Zhuhai, China; ^4^School of Life Sciences, Sun Yat-sen University, Guangzhou, China

**Keywords:** NF-κB pathways, shrimp, bacteria, white spot syndrome virus (WSSV), Toll-like receptor, IMD

## Abstract

The outbreak of diseases ordinarily results from the disruption of the balance and harmony between hosts and pathogens. Devoid of adaptive immunity, shrimp rely largely on the innate immune system to protect themselves from pathogenic infection. Two nuclear factor-κB (NF-κB) pathways, the Toll and immune deficiency (IMD) pathways, are generally regarded as the major regulators of the immune response in shrimp, which have been extensively studied over the years. Bacterial infection can be recognized by Toll and IMD pathways, which activate two NF-κB transcription factors, Dorsal and Relish, respectively, to eventually lead to boosting the expression of various antimicrobial peptides (AMPs). In response to white-spot-syndrome-virus (WSSV) infection, these two pathways appear to be subverted and hijacked to favor viral survival. In this review, the recent progress in elucidating microbial recognition, signal transduction, and effector regulation within both shrimp Toll and IMD pathways will be discussed. We will also highlight and discuss the similarities and differences between shrimps and their *Drosophila* or mammalian counterparts. Understanding the interplay between pathogens and shrimp NF-κB pathways may provide new opportunities for disease-prevention strategies in the future.

## Introduction

Shrimp farming is an important economic activity in China and many Southeast Asian countries, such as the Philippines, Indonesia, Malaysia, Thailand, and Vietnam, and it provides an important contribution to the diversity of income strategies for a large proportion of people living in these countries. According to the statistics of the Food and Agriculture Organization (FAO) of the United Nations, seawater and low-salinity brackish-water shrimp-culture production in the world expanded from 1,325 metric tons in 1950 to 4,875,793 metric tons in 2015. The main species of shrimp aquaculture include *Litopenaeus vannamei (L. vannamei*), *Fenneropenaeus chinensis* (*F. chinensis*), *Penaeus monodon* (*P. monodon*), *Marsupenaeus japonicus* (*M. japonicus*), *Procambarus clarkii* (*P. clarkii*), and *Macrobrachium rosenbergii* (*M. rosenbergii*) (1). Among them, *L. vannamei* accounted for nearly 80% of global shrimp production in 2015. Even though the production of shrimp has increased considerably in recent years, emerging diseases have been the main threat to restrict the sustainable development of the industry worldwide. A wide range of pathogens, including viruses, bacteria, fungi, and parasites, can infect shrimp, among which the white spot syndrome virus (WSSV) and *Vibrio parahaemolyticus* (VP_AHPND_) have led to the most serious economic losses in the shrimp-cultivation industry worldwide. WSSV is the causative agent of white spot syndrome (WSS), which causes 100% mortality within 7–10 days (2). VP_AHPND_ contains two 69-kb plasmid-carrying binary Pir-like toxin genes, ${\text{Pir}}_{-}^{\text{vp}}$A and ${\text{Pir}}_{-}^{\text{vp}}$B, which are the causative agents of acute hepatopancreatic necrosis disease (AHPND) (3, 4). Outbreak of diseases often occurs when the homeostasis between pathogens and host resistance is disrupted. Therefore, better understanding the shrimp immune system will provide more effective strategies for disease prevention and control under specific culture ecosystems.

Innate immunity is characterized by the activation of pattern-recognition receptors or proteins (PRRs or PRPs) capable of sensing and binding with pathogen-associated molecular patterns (PAMPs) that are only presented in the pathogen but not in the host (5). The innate immune system of invertebrates is generally divided into humoral defenses and cellular defenses. Humoral defense include the production of soluble effector molecules, such as antimicrobial peptides (AMPs), while cellular defenses, such as phagocytosis and encapsulation, are mediated by circulating hemocytes (6–8). Like other invertebrates, shrimp mainly rely on the innate immune response to control and clear invading pathogens following infection. Upon PRRs-PAMPs interaction, the cellular and humoral immune responses are rapidly activated, which adopt a coordinated strategy to eliminate foreign pathogens. The nuclear factor-κB (NF-κB) family of transcription factors comprise key regulators of humoral immune responses that are indispensable for the host-defense system (9). In shrimp, there are two NF-κB transcription factors, namely Dorsal and Relish (10–14). Dorsal is the critical transcription factor in the Toll signaling pathway, while Relish plays a key role in the IMD signaling pathway (10–14). Since the first shrimp Toll receptor, LvToll or LvToll1, was reported in 2007 (15, 16) and the first shrimp IMD homolog, LvIMD, was identified in 2009 (17), a series of significant findings have been made over the last 20 years. Increased attention has been focused on the identification of pathway components and deciphering the molecular mechanisms underlying the Toll and IMD pathways related to infection. Herein, we present an overview of our current knowledge of the two NF-κB pathways in immune responses to bacterial and WSSV infection, with the hope of providing novel insights into the immune system of shrimp.

## The Toll Pathway

Tolls and Toll-like receptors (TLRs, usually defined from the Toll homologs of vertebrates) have been recognized as major PRRs in innate immunity and they play an indispensable role in recognition of microbes during host defense (18, 19). In *Drosophila*, a total of nine Toll receptors have been identified (20). *Drosophila* Toll-1, or simply Toll, was the first Toll identified (21), and its mediated cellular-signaling cascades, comprising the MyD88/Tube/Pelle/Cactus/Dorsal axis, are widely considered to form the canonical Toll pathway. The *Drosophila* Toll pathway can be induced by Gram-positive bacteria, fungi, and some viruses (20). The activation of Toll leads to the initiation of cellular-signaling transduction and ultimately results in the systemic production of specific antimicrobial peptides (AMPs), such as the antifungal peptide Drosomycin, which is widely considered as a conventional readout of activation of the *Drosophila* Toll pathway (22, 23). After the identification of the *Drosophila* Toll pathway, many findings have been obtained in vertebrates, especially mammals, and some invertebrates, including shrimp. In recent years, many components of the shrimp canonical Toll pathway and its concomitant regulators—including Spätzle, Toll, MyD88, Tube, Pelle, Pellino, TRAF6, Dorsal, Cactus, Tollip, SARM, Flightless-I, and β-arrestin—have been identified and functionally characterized. These characterizations have provided some surprising insights into the shrimp Toll pathway in the context of receptor sensing, signaling transduction, and host-pathogen interactions. The identified components of the Toll-related pathway in penaeid shrimp are listed in Table 1.

**Table 1:** Components of canonical Toll signaling pathway identified in shrimps.

**Components**	**Species**	**Gene names**	**Accession numbers**	**In response to pathogenic infections**	**References**
Toll	*L. vannamei*	LvToll1	DQ923424.1	Gill: *M. lysodeikticus, V. harveyi, V. anguillarum*, and WSSV, up	(15, 16, 24, 25)
		LvToll2	JN180637	Gill: WSSV, up	(26)
		LvToll3	JN180638	Gill: WSSV, up	(26)
		LvToll4	Unsubmitted	Gill, Hemocytes: WSSV, up	(27)
		LvToll5	Unsubmitted	ND	(27)
		LvToll6	Unsubmitted	ND	(27)
		LvToll7	Unsubmitted	ND	(27)
		LvToll8	Unsubmitted	ND	(27)
		LvToll9	Unsubmitted	ND	(27)
	*P. monodon*	PmToll	GU014556.1	ND	(28, 29)
		PmToll9	KY438975.1	ND	(30)
	*F. chinensis*	FcToll	EF407561	Lymphoid organs: *V. anguillarum*, up; WSSV, down	(31)
	*M. japonicus*	MjToll1	AB333779.1	ND	(32)
		MjToll2	AB385869.1	ND	Direct Submission
	*M. rosenbergii*	MrToll	JF895474	Hemocytes: *Aeromonas caviae*, up	(33)
		MrToll	KX610955.1	Gill: WSSV, up	(34)
		MrToll1	KJ188410.1	ND	Direct Submission
		MrToll2	KJ188411.1	ND	Direct Submission
		MrToll3	KJ188412.1	ND	Direct Submission
	*P. clarkii*	PcToll	KP259728.1	Hemocytes, Hepatopancreas, Gill, Intestine: *S. aureus* and *Vibrio*, up	(19)
		PcToll1	KP259728		(19)
		PcToll2	KX505307	Hemocytes: *Vibro* 0-12 hpi up, 12-48 hpi down	(35)
		PcToll3	KU680805.1	Hemocytes: *Vibrio* and WSSV, up	(36)
		PcToll4	KU680806.1	Intestine: WSSV, up	(37)
		PcToll5	KU680807.1	ND	Direct Submission
Spätzle	*L. vannamei*	LvSpz1	JN180646	Gill: *V. alginolyticus* and WSSV, up	(26)
		LvSpz2	JN180647	Gill: *V. alginolyticus*, down	(26)
		LvSpz3	JN180648	Gill: *V. alginolyticus* and WSSV, up	(26)
		LvSpz4	KX060799	Gill: *S. aureus, V. alginolyticus*, up	(38)
	*P. monodon*	PmSpätzle 1	KY053796	hemocytes: *S. aureus, V. harveyi* and WSSV, up	(39, 40)
		PmSpätzle2	KY053798	ND	(39)
		PmSpätzle3	KY053797	ND	(39)
	*F. chinensis*	Fc-Spz	EU523114.1	Hemocytes, Heart, Hepatopancreas, Gill, Stomach, Intestine: *V. anguillarum* and WSSV, up	(41)
	*M. japonicus*	MjSpz	KX424932	Gill: WSSV, up	(42)
	*M. rosenbergii*	MrSpz	Unsubmitted	Hemocytes: *A. caviae*, up	(43)
MyD88	*L. vannamei*	LvMyD88	JX073568.1	Hemocytes: *S. aureus, V. parahaemolyticus* and WSSV, up	(44)
		LvMyD88-1	JX073567.1	ND	(44)
	*P. monodon*	PmMyD88	KJ577578.1	Hemocytes, Lymphoid organ, Gill, Stomach, Hepatopancreas, Midgut, Hindgut: WSSV, up	(45)
	*F. chinensis*	FcMyD88	JX501341.1	Hemocytes: *M. lysodeikticus, V. anguillarium*, up; WSSV, up	(46)
Tube	*L. vannamei*	LvTube	JN180645.1	Gill, Hepatopancreas: *V. alginolyticus* and WSSV, up, Intestine: *V. alginolyticus* and WSSV, down	(47)
		LvTube-1	KC346865	Hemocytes: *S. aureus, V. parahaemolyticus* and WSSV, up	(48)
	*P. monodon*	PmTube	KR136276.1	Hemocytes: WSSV, up	(49)
Pelle	*L. vannamei*	LvPelle	KC346864	Hemocytes: *S. aureus, V. parahaemolyticus* and WSSV, up	(48)
TRAF6	*L. vannamei*	LvTRAF6	HM581680.1	Intestine: *V. alginolyticus* and WSSV, down; Gill, Hepatopancreas: *V. alginolyticus* and WSSV, up	(50)
	*P. monodon*	PmTRAF6	KJ577579.1	Hemocytes, Lymphoid organ, Stomach, Hepatopancreas: WSSV, up; Gill, Midgut, Hindgut: WSSV, down	(45)
	*F. chinensis*	FcTRAF6	JQ693681.1	ND	(51)
IKKβ	*L. vannamei*	LvIKKβ	AEK86518	Gill, Hepatpancreas, Intestine: *V. alginolyticus* and WSSV, up; Hemocytes: *V. alginolyticus* and WSSV, down	(52)
Dorsal	*L. vannamei*	LvDorsal	FJ998202.1	Gill: WSSV, up	(11, 53)
	*F. chinensis*	FcDorsal	EU815056.1	Hemocytes, Lymphoid: *M. lysodeikticus, V.anguillarium*, up; Hemocytes: WSSV: 1 hpi up, 2–5 hpi down; Lymphoid: WSSV: 2 hpi up, 3–14 hpi down	(13)
	*M. japonicus*	MjDorsal	KU160503.1	Gill, Hemocytes, Intestine: *S. aureus*, up	(54)
	*M. rosenbergii*	MrDorsal	KX219631.1	Gill: WSSV, up	(10)
Cactus	*L. vannamei*	LvCactus	JX014314.1	Hemocytes: *S. aureus, V. parahaemolyticus*, up; WSSV, down	(55)
	*F. chinensis*	FcCactus	JQ693681	Hemocytes: *M. lysodeikticus, V. anguillarium*, up	(51)
Tollip	*L. vannamei*	LvTollip	JN185616.1	Hepatopancreas, Gill, Intestine: *V. alginolyticus* and WSSV, up, Hemocytes: *V. alginolyticus*, down; WSSV, up	(56)
Pellino	*L. vannamei*	LvPellino	KC346863.1	Hemocytes: *S. aureus, V. parahaemolyticus* and WSSV, up	(57)
SARM	*L. vannamei*	LvSARM	JN185615	Hemocyte, Gill, Intestine: *V. alginolyticus* and WSSV, up; Hepatopancreas: *V. alginolyticus*, down; WSSV, up	(58)
Flightless-I	*L. vannamei*	LvFli-I	KC800820	Hemocytes: *S. aureus, V. parahaemolyticus*, up; WSSV, down	(59)
β-arrestin	*M. japonicus*	Mjβarr1	KU160500	ND	(54)
	*M. japonicus*	Mjβarr2	KU160501	ND	(54)
ATF	*P. clarkii*	PcATF4	KX505308	Hemocytes: *Vibro*, up	(35)

*up, differentially up-regulated; down, differentially down-regulated; ND, Not determined*.

### Toll and Spätzle

**Figure 1:**
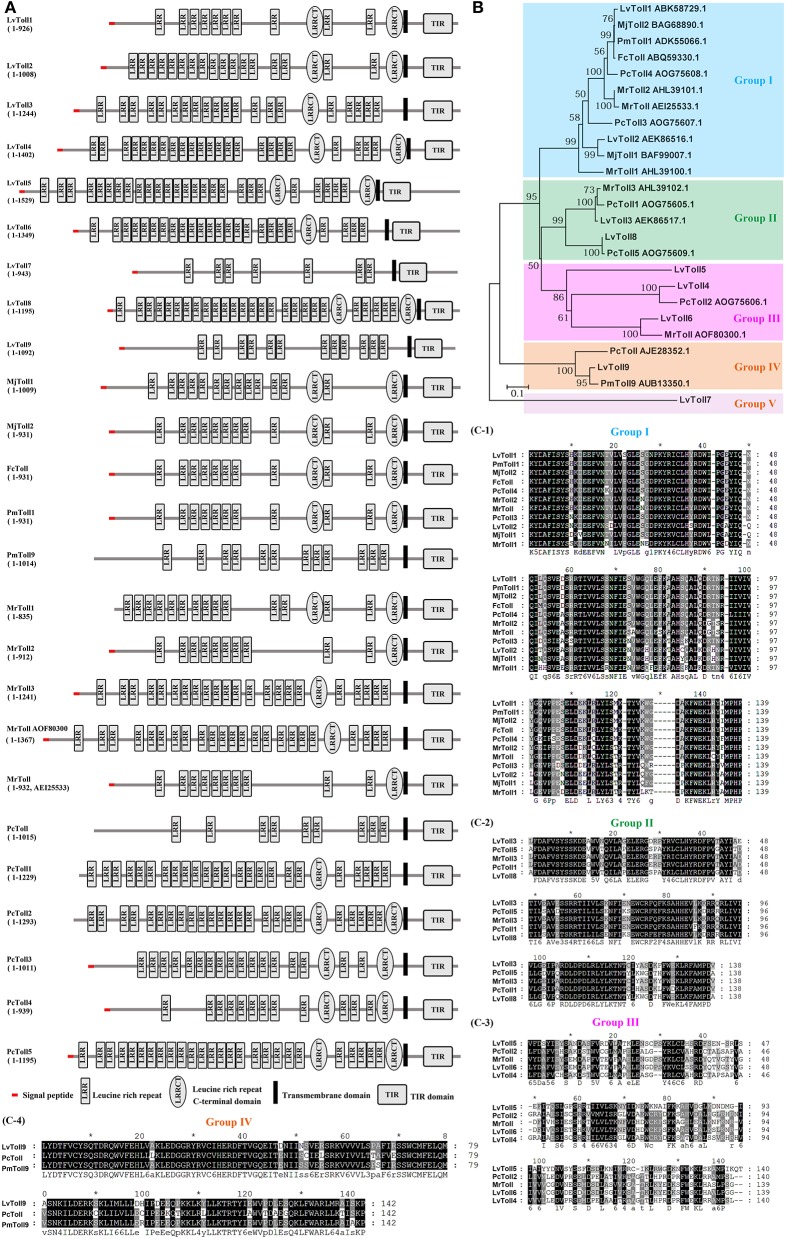
Tolls identified from shrimp. **(A)** Architectural representation of characteristic domains of 25 Tolls from six different shrimp species. **(B)** Phylogenetic-tree analyses of TIR domains of the Tolls. Genbank accession numbers of some Tolls are shown after their scientific names. **(C)** Multiple sequence alignments of shrimp Toll proteins from Group I (C-1), Group II (C-2), Group III (C-3), and Group IV (C-4).

Tolls and TLRs are characterized by an extracellular domain containing various numbers of leucine-rich repeats (LRRs) and a cytoplasmic-signaling domain, Toll/IL-1R (TIR), that can interact with cytoplasmic-adaptor molecules, thereby activating downstream-signaling events (60). To date, 25 genes encoding distinct Toll homologs have been identified in shrimp from six different species including LvToll1-9 from *L. vannamei* (15, 16, 24–26, 61), FcToll from *F. chinensis* (31), PmToll1, and PmToll9 from *P. monodon* (28–30), MjToll1-2 from *M. japonicas* (32), PcToll and PcToll1-5 from *P. clarkii* (19, 35–37), and MrToll1-3 and two MrTolls from *M. rosenbergii* (33, 34) (Figure 1A). A phylogenetic tree based on TIRs of the 25 Tolls has been constructed, from which we observe that Tolls from shrimp can be divided into five groups (Figure 1B). Group I contain the majority of Tolls identified, with up to a total of 11 Tolls from all 6 shrimp species. Groups II, III, IV, and V contain five, five, three, and one Toll(s) from partial shrimp species, respectively (Figure 1B). Alignment of TIR domains in each corresponding group indicates that the TIR sequences in each group are highly conserved (Figure 1C). In addition to the shared LRRs in the ectodomain, Tolls usually possess one or two Leucine-rich-repeat C-terminal domains (LRRCTs) and/or a signal peptide in the N-terminal (Group I–III). In comparison with other Tolls, PmToll9, and PcToll clustered in Group IV have no LRRCT or signaling peptide (19, 30). The conserved TIR domains in each group suggest that they might focalize the same kind of adaptor molecules, suggesting that they could activate the same signaling pathway. However, in consideration that LRR-LRRCT ectodomains of Tolls function as ligand-recognition sites (62), it is likely that Tolls—even in the same group that harbor conserved cytoplasmic TIR domains but exhibit a variety of structures in the ectodomain—could recognize some specific ligands and respond to different pathogens. This may be in line with the observation that distinct Tolls in different species respond to diverse bacterial and viral infections but converge on the induction of the same kind of effectors, such as AMPs.

So far, the Tolls from *L. vannamei* are still the most studied Tolls in shrimp. LvToll1, LvToll2, and LvToll3 in gill are all up-regulated during WSSV infection, while only LvToll1 expression increases during *Vibrio* infection (16, 24–26). LvToll2 could significantly induce the activation of NF-κB pathway controlling AMPs in the background of insect cells, whereas these AMPs appear to be not induced by LvToll1 and LvToll3 (15, 26, 63). Further, knockdown of LvToll1 by RNA interference (RNAi) does not influence the susceptibility of shrimp to WSSV infection, but silencing of LvToll1 significantly increases the mortality of shrimp and reduces bacterial clearance after challenge with *Vibrio harveyi* (16, 64). In a recent study, another six Tolls of LvToll4–9 have been cloned, and the authors found that eight Tolls, except for Toll2, exhibit different levels of resistance to WSSV. Furthermore, LvToll4 has been shown to be a key factor (possibly as a receptor) for sensing WSSV and therefore activate the downstream Dorsal, resulting in the inducing of specific AMP production (27). As for other species, FcToll in lymphoid is significantly induced after 5-h-post *Vibrio* challenge but its expression markedly is reduced immediately after WSSV exposure (31). MrToll, PcToll1, and PcToll2 are up-regulated after bacterial infection, while PcToll4 can regulate the expression of AMPs to defend against WSSV (19, 33, 35, 37). Moreover, PcToll3 in hemocytes responds to both bacterial and WSSV infection (36). Although the RNAi phenotypes of most Tolls are unknown, their expression is responsive to bacterial or viral infection; it is therefore tempting to speculate that they are involved in host defense.

The subcellular localization of TLRs could somewhat influence PAMP accessibility (65). Some mammalian TLRs localized on cell surfaces, including TLR1, TLR2, TLR4, TLR5, TLR6, and TLR11, could recognize mainly microbial-membrane components, such as lipids, proteins, and lipoproteins, while there are also intracellularly expressed TLRs including TLR3, TLR7, TLR8, and TLR9, located in the endosome and mainly recognize microbial nucleic acids (65–67). Through ectopic expression in *Drosophila* S2 cells, LvToll1, and LvToll3 have been shown to localize at both the membrane and cytoplasm, while LvToll2 is ubiquitously distributed within the cytoplasm (15, 26). Over-expression of PmToll9 with GFP tag in Hela cells showed that PmToll9 is mainly located in the cytoplasm (30). Furthermore, more direct detection of cellular localization of shrimp Tolls should be conducted *in vivo* using immunofluorescent staining or immunohistochemical staining by using Toll-specific antibodies. Whether the shrimp Tolls localization is implicated with their potential roles in immune-signaling pathways remains unknown.

Different from mammalian TLRs, Toll in *Drosophila* cannot recognize PAMPs directly but need the cytokine-like molecule, Spätzle (Spz) as a ligand (45, 64, 68). Spätzle is a member of the cysteine-knot protein superfamily, which was cleaved from an inactive pro-Spätzle. Pro-Spätzle contains the pro-domain that prevents Spätzle from binding to the Toll receptor, via the serine protease, Easter, during dorsoventral patterning or Spätzle-processing enzymatic activity upon infection. The activated *Drosophila* Spätzle contains 106 residues in the C-terminal domain (C-106), which is sufficient to active the Toll pathway (41, 69). The interaction between Spätzle C-106 dimer and the extracellular domain of Toll rearranges cytoplasmic TIR domains conformation, thereby generating a docking site for recruiting TIR-domain-containing adaptors—such as MyD88, which in turn activate the Toll pathway (70). To be noted, Toll7 in *Drosophila* has been considered as a specific PRR for sensing vesicular-stomatitis viruses, because that it can bind to the plasma membrane of this virus (71). Surprisingly, several Tolls from shrimp are reported to detect some PAMPs directly. For example, *in vitro*, Toll1 and Toll3 from *L. vannamei* can combine with CpG ODN 2395 (72). Additionally, three Tolls from *M. japonicas* can directly interact with both PGN and LPS (73). Interestingly, two Tolls from *M. japonicas* are homologous to the above Toll1 and Toll3 from *L. vananmei* (73). These data indicate that in shrimp, one type of Toll could sense multiple types of PAMPs, which are more similar to TLRs in mammals.

The first Spätzle-like protein from shrimp was identified in *F. chinensis* in 2009 and was named as FcSpz (41). The activated form of FcSpz requires a seven cysteine residues on C-terminal which is essential for intra-molecular and inter-molecular disulfide bonds to form Spätzle homodimers (41). FcSpz respond to *Vibro alginolyticus* and WSSV infection, and the injection of C-terminal active FcSpz domain (114 residues) *in vivo* could activate the promoter of shrimp AMP Crustin2 (41). In addition to FcSpz, seven more Spätzles have been cloned and identified in different shrimp, including LvSpz1-4, PmSpz1-3, MjSpz, and MrSpz (26, 38, 39, 42, 43). All of these Spätzle genes can respond to bacterial and/or WSSV infections. Interestingly, multiple Spätzles—such as LvSpz1, LvSpz2, LvSpz3, and FcSpz3—lack the seventh Cys residue that is important to disulfide-linked homodimer formation, and that favors its binding to Toll receptors, which might explain their slight effects on induction of AMP expression (26, 41). Instead, the Spätzle domain of LvSpz4 containing the seventh Cys residue can strongly activate the NF-κB pathway that regulates AMPs such as Penaeidin 4 (PEN4), Drosomycin (Drs), Attacin (Atta), and Metchnikowin (Mtk) in *Drosophila* S2 cells. Moreover, the activating transcription factor 4 (ATF4) and X-Box-binding protein 1 (XBP1), components of the unfolded protein response (UPR), are capable of inducing the expression of LvSpz4, which suggests that LvSpz4 could be a regulator to link the Toll-NF-κB pathway and the UPR (38). Although a series of Tolls and Spzs have been identified, there are few studies that have investigated the interactions among these proteins. Detecting the interplay between Spz and Toll will be helpful to illustrate which pairs of Spzs/Tolls are function to mediate the shrimp Toll pathway inactivation, and induce immune-related genes expression.

### Cascades of the Toll Pathway

**Figure 2:**
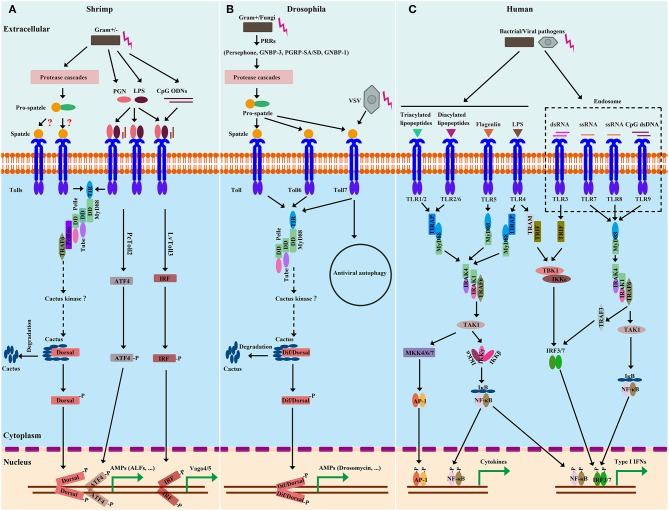
Comparison of Toll and TLR pathways from shrimp **(A)**, *Drosophila* **(B)**, and *Human* **(C)**. Homologies between signaling components are depicted by similar shapes and colors. In shrimp, there are two extracellular-signaling routes leading to Toll pathway activation. Considering that many Spätzle (Spz) genes from shrimp have been identified and are able to induce Toll-Dorsal-controlled AMPs, the extracellular cleavage of Spz mediated by protease cascades could be similar to those of *Drosophila*. In the immune responses to microbial recognition, the protease cascades lead to the activation of Spz-processing enzymes (SPEs) to cleave full length Spz. Upon proteolytical processing, the Spz prodomain is cleaved, exposing the C-terminal Spz parts that are critical for binding of Toll. Spz binding to the Toll receptor initiates intracellular signaling. In contrast, shrimp Tolls are able to sense and directly bind to some conserved motifs, such as PGN, LPS, and ODN, from microbes, which are similar to those of *Human*. Upon binding to these motifs, shrimp Tolls are activated and initiate intracellular signaling. In the intracellular-signaling event, signaling cascades of shrimp Toll pathways broadly resemble those of *Drosophila*. After Toll activation, the adaptor MyD88 builds a signaling complex with Tube and Pelle. The MyD88-Tube-Pelle complex in turn recruits other regulators, such as Pellino and TRAF6, which leads to the phosphorylation and degradation of Cactus and thereby releases Dorsal (and/or Dif in *Drosophila*) to translocate to the nucleus and activate transcription. In *Human*, there are MyD88-dependent and MyD88-independent signal-transduction events. The intracellular signaling of *Human* can lead to active NF-κB, AP-1 and IFN regulatory factor 3/7 (IRF3/7) for their nuclear translocation and subsequent transcriptional activation of target genes. Of note, Toll3 from *L. vannamei* and Toll2 from *P. clarkii* have been shown to activate IRF and ATF4, respectively, leading to transcriptional synthesis of some antiviral effectors, such as Vago4/5 and ALFs.

The cascades of the Toll pathway have been well studied in both flies (e.g., *Drosophila*) and mammals (e.g., *Human*). In *Drosophila*, the Toll pathway can respond to Gram-positive bacteria, fungi, and some viruses (20). The activation of Toll triggers intracytoplasmic TIR domains dimerization, therefore recruiting the adaptor MyD88 via its own TIR domain (74). The second adaptor protein, Tube, binds with MyD88, and the protein kinase Pelle; these two interaction is formed via pairwise interactions of death domains (Figure 2) (75, 76). Pelle is able to phosphorylate itself, and the autophosphotylation results in the inhibitor of κB, Cactus, phosphorylation and destruction as well as the phosphorylation of the homolog of tumor necrosis factor receptor-associated factor 6 (TRAF6), dTRAF2. Then, depending on the context, the transcription factor, Dif, or Dorsal, then translocate from cytoplasm to nucleus (20, 77).

In the TLR signaling pathway in *Human*, after binding with their ligands, all TLRs except for TLR3 initiate the MyD88-dependent pathway through recruiting MyD88 via the TIR domain (78). Similar to that of *Drosophila*, in *Human*, the signaling complex is composed of MyD88, the Tube ortholog, IRAK4, and the Pelle ortholog, IRAK1 (79, 80). The phosphorylated IRAK-1 gets associated with TRAF6 (81), resulting in the disassociating of IRAK1/TRAF6 complex from the above receptor complex, instead interacting with another complex consisting of transforming growth factor (TGF)-β activated kinase 1 (TAK1) and TAK1 binding protein (TAB) 1, TAB2 and TAB3 (57, 82). Pellino is a highly conserved E3-class ubiquitin ligase. It binds to the phosphorylated IRAK1, resulting in K63 polyubiquitination of IRAK1. The polyubiquitinated IRAK1 interacts with the ubiquitin-binding domain of NF-κB essential modifier (NEMO, also named IKKγ) (57, 83). This interaction leads to the TAK1-TAB1-TAB2 complex and IKKγ-IKKα-IKKβ complex into close proximity, which subsequently results in TAK1-mediated phosphorylation and activation of the IKKs (84). The activated IKKs phosphorylates the NF-κB cytoplasmic inhibitory protein, IκB, leading to polyubiquitylation and degradation of IκB and release of NF-κB from the IκB/NF-κB complex (57, 85). NF-κB subsequently undergoes nuclear translocation, where it induces the expression of a wide range of immune-modulatory genes, including pro-inflammatory cytokines. In the TIR-domain-containing adaptor protein-inducing IFN-β (TRIF)-dependent pathway, after recognizing dsRNA, TLR3 recruits TRIF, TRAF6, and TRAF3, leading to the activation of IKK-related kinases, including TANK-binding kinase I (TBK1) and IKKε, thereby resulting in the activation of IRF3/7 signaling pathways that eventually induce the transcription of type-I interferon (IFN; Figure 2) (77, 86). Of note, TLR4, located on the cell surface, can activate both of the distinct intracellular signaling pathways via the adaptor molecules, MyD88 and TRIF, that finally results in NF-κB and MAPK activation to trigger the expression of pro-inflammatory cytokines and/or lead to IRF3/7 activation to induce Type-I IFNs production (Figure 2) (87, 88).

**Figure 3:**
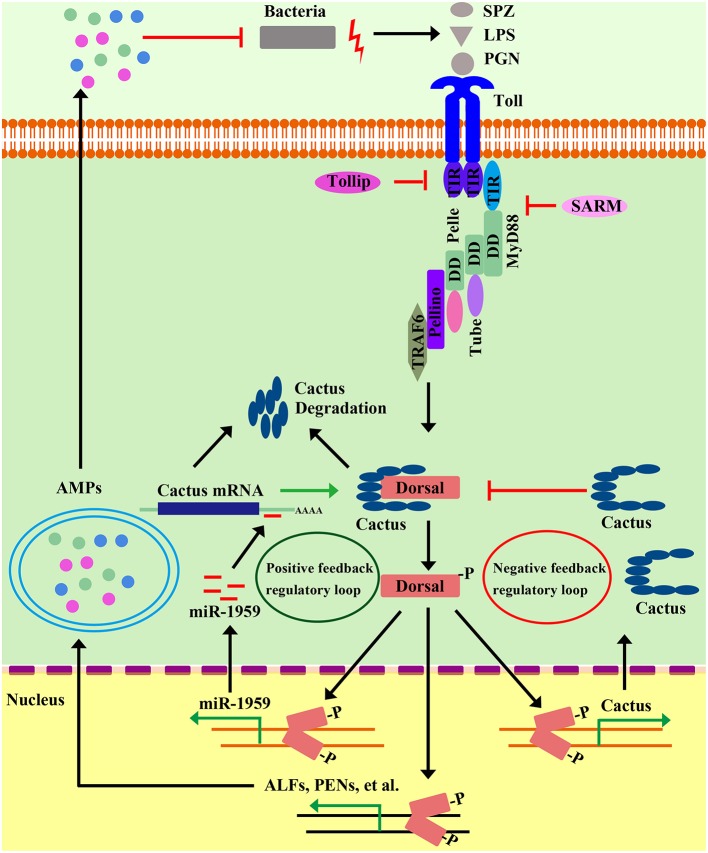
Positive and negative regulatory events in shrimp Toll pathways. Positive regulation of the shrimp Toll pathway can amplify signaling cascades to rapidly limit and clear invading pathogens. In microbial recognition, intracellular signaling leads to activation of Dorsal, which in turn induces the transcription of a microRNA gene, miR1959. MiR1959 can target the mRNA of Cactus, an inhibitor of Dorsal, resulting in reduced Cactus protein levels, which leads to the up-regulation of Dorsal activity to boost some effectors, such as AMP expression against invading microbes. On the other hand, the activation of the Toll pathway must be controlled and inhibited to ensure a properly timed and adjusted response. Along with the clearing of invading pathogens, the gradually increased Cactus leads to the inhibition of Dorsal activity by their direct interaction, which establishes a negative-feedback loop to abate NF-κB pathway signaling to avoid the sustained activation of this pathway. In addition, there multiple negative regulators have been identified—including Tollip, SARM, FliI, and β-Arrestin—that modulate the Toll pathway in different ways.

Beginning with Toll, most components of the *Drosophila* Toll pathway have shrimp homologs (Table 1). Four MyD88s in three species (LvMyD88, LvMyD88-1, PmMyD88, and FcMyD88) (44, 46), three Tubes in two species (LvTube, LvTube-1, and PmTube) (48, 49), and LvPelle (47, 48) have been identified in shrimp. Each of these proteins contains a protein-interaction motif named death domain, which is originally described in apoptotic pathways (89). LvPellino has been the sole Pellino homolog found in shrimp until recently, and is able to interact with *L. vannamei* Pelle (LvPelle) and positively regulate the activity of LvDorsal (57). Three TRAF6s (LvTRAF6, PmTRAF6, and FcTRAF6) have been characterized in three species with a RING-type zinc-finger domain in the N-terminal, followed by two TRAF-type zinc-finger domains, a coiled region and a MATH domain in the C-terminal (45, 50, 51). So far, no Dif homolog has been found in shrimp, while four Dorsal proteins (LvDorsal, FcDorsal, MjDorsal, and MrDorsal) in shrimp are similar in sequence to the mammalian NF-κB (p65) and *Drosophila* Dorsal (10, 11, 13, 54). However, *Drosophila* Dorsal mainly responds to Gram-positive bacteria but does respond to a few Gram-negative bacteria (20, 64), whereas shrimp Dorsal—such as LvDorsal and FcDorsal—appear to respond to both Gram-positive and Gram-negative bacteria, as well as viruses such as WSSV (11, 13). In both *Drosophila* and shrimp, each Dorsal contains a conserved Rel-homology domain, which is a bearing sites for DNA binding, dimerization, and interaction with an inhibitor. The Cactus or its ortholog, IκB, function as an inhibitor of Dorsal (NF-κB), contain an N-terminal regulatory region responsible for ubiquitin recognition and proteasomal degradation, and have neighboring ankyrin repeats that are capable of binding with the Rel-homology region and a destabilizing C-terminal PEST domain that are required for inhibition of DNA binding (90). All three domains are found and conserved in LvCactus and FcCactus, suggesting that shrimp Cactus could comply with a similar function and regulatory fashion to those of *Drosophila* Cactus (51, 55). In addition to these classical pathway components, several members involved in regulating the Toll pathway directly or indirectly have also been identified including activating transcription factor 4 (PcATF4) (35), Tollip (LvTollip) (56), Flightless-I (LvFli-I) (59), β-arrestins (Mjβarr1-2) (54), and sterile-alpha and armadillo motif-containing protein (LvSARM) (58) (Figure 3 and Table 1).

Apart from Dorsal, the transcription factor of classical Toll pathway, in *P. clarkii*, PcATF4 is involved in PcToll2 signaling to promote AMP expression, which suggests that PcATF4 might be another crucial transcription factor involved in crustacean Toll signaling to prevent shrimp from Gram-negative bacterial invasion (35). Likewise, there is another case in which the Spätzle, LvSpz4, has been reported to be regulated by ATF4, one of the major components of the UPR pathway (38). Although there is no ATF4 homolog that has been found in *Drosophila* yet, in human, ATF4 has also been shown to participate in TLR4 pathway to defend against Gram-negative bacterial invasion by promoting inflammatory cytokines secretion, through working together with another transcription factor, c-Jun (36, 91). Similar to mammal TLR3 inducing IRF3/7 activation, LvToll3 is also involved in the induction of IRF-Vago-JAK/STAT pathway related genes, suggesting that LvToll3-mediated intracellular signaling may follow a cascade akin to those of vertebrates (Figure 2) (61, 92). These observations demonstrate that the canonical Toll pathway might crosstalk with other signaling pathways in a coordinated manner to launch a specific immune response.

### Negative Regulators of the Toll Pathway

The Toll pathway is an important part of the innate immune system, but hypernomic activation of the Toll pathway can cause immune disorder and seriously affect the health, even the survival of an organism. So, the fine-tuned and subtle regulation—including negative regulators of the Toll pathway—has evolved to keep the balance of immunity (Figure 3). The IκB is the evolutionarily conserved inhibitor of canonical NF-κB pathway. As mentioned in above section, degradation of the NF-κB inhibitor, IκB, allows NF-κB free from the cytoplasmic NF-κB/IκB complex, and the freed NF-κB migrates from cytoplasm to nucleus to induce various target genes (93). Cactus is the homolog of IκB in invertebrates and mediates both negative- and positive-feedback regulatory loops of NF-κB via different pathways in shrimp. The induction of LvCactus is activated by LvDorsal but on the other hand is inhibited by LvCactus itself (55). LvCactus interacts with LvDorsal, which is confirmed by immunoprecipitation assays and fluorescent microscopy (55). Four of the five putative NF-κB-binding motifs in the promoter of LvCactus have been shown to be targeted by LvDorsal (55). Hence, we hypothesize that the Toll pathway is activated in some cases by bacterial infection, following by rapid up-regulation of LvDorsal activity that induces massive effectors that function against invaders. Additionally, LvDorsal activation also contributes to the increased expression of LvCactus, which feeds back to inhibit LvDorsal activity via their direct interactions. These interactions create a negative-feedback loop to abate NF-κB-pathway signaling in order to avoid the sustained activation of this pathway (Figure 3) (55). There is also a positive-feedback regulatory loop in the Toll pathway with the participation of Dorsal, Cactus, and a host microRNA, miR-1959 (94). Dorsal can directly bind the NF-κB-binding motif in the promoter region of miR-1959 and activate its transcription and, in turn, miR-1959 targets the 3′-untranslated region of Cactus, reducing the protein level of Cactus, further leading to enhanced activation of Dorsal (Figure 3) (94).

Additionally, Sun et al. showed that in the kuruma shrimp, *M. japonicus*, β-arrestin can negatively regulate Toll signaling in two different ways. β-arrestin can prevent Dorsal translocation via β-arrestin-Cactus-Dorsal heterotrimeric complex with Cactus as the bridge. β-arrestin and Dorsal do not come into contact with each other; instead, the ankyrin-repeat domain and C-terminal PEST domain of Cactus separately bind the arrestin-N domain of β-arrestin and RHD domain of Dorsal, respectively (54). After formation of the oligomeric β-arrestin-Cactus-Dorsal complex, Cactus phosphorylation, and degradation is prevented, which inhibits Dorsal translocation into the nucleus as well as the activation of Toll signaling pathway. On the other hand, β-arrestin can inhibit Dorsal phosphorylation and transcriptional activity. Extracellular signal-regulated protein kinase (ERK) can function as a kinase with a capacity for Dorsal phosphorylation. Meanwhile, β-arrestin can interact with non-phosphorylated ERK through its arrestin-C domain to inhibit ERK phosphorylation, which affects Dorsal phosphorylation and thus inhibits its transcriptional activity and nuclear localization (54).

In addition to these regulators mentioned above that target the transcription factor Dorsal or its inhibitor Cactus, Toll-interacting protein (Tollip) from *L. vannamei* functions as a negative modulator in the Toll pathway through interacting with up-stream Toll receptors (56). Tollip associates directly with some of the Tolls or TLRs through TIR-domain-mediated interactions and therefore inhibits Toll/TLR-mediated NF-κB activation by suppressing adaptor proteins, such as IRAK1, phosphorylation, and kinase activity (56). So far, no Tollip-like homolog has been found in the *Drosophila* genome. However, forcible expression of LvTollip in *Drosophila* S2 cells significantly inhibits the promoter activities of the Toll pathway controlling the antifungal-peptide gene, Drosomycin (Drs). In *Human* HEK 293T cells, LvTollip has been demonstrated to significantly suppress the inductions of both NF-κB and IFN-β (56). These findings might suggest that from shrimp to humans, the Tollips are functionally conserved in the TLR-NF-κB signaling pathway.

Mammalian MyD88 is known as a universal adaptor protein in the downstream signaling of many different kinds of TLRs, with the exception of TLR3, which instead recruits TRIF (56, 95). Hence, MyD88 is regarded as a perfect target for immunity regulation. Flightless-I (FliI) was originally characterized as a gene mutation that causes defects in the flight muscles in *Drosophila melanogaster* (96). The FliI protein belongs to the gelsolin superfamily of actin-remodeling proteins that usually contain six C-terminal gelsolin-like domains (GEL), and harbors multiple unique LRR domains in the N-terminal responsible for protein-protein or protein-lipid interactions (97). FliI is widely identified as a negative regulator to modulate NF-κB activity through interfering with MyD88-Toll receptor interactions (98). LvFliI, identified from *L. vannamei*, is up-regulated *in vivo* in response to the challenges of LPS, Poly (I: C), CpG-ODN 2006, *V. parahaemolyticus, Staphyloccocus aureus*, and WSSV, and it is shown to suppress the expression of the NF-κB-pathway-dependent AMPs, including LvPEN2, LvCrustin, LvALF1 (anti-LPS factor), and LvLyz1 (Lysozyme) *in vivo* (59). Also, over-expression of LvFliI in *Drosophila* S2 cells can negatively regulate the promoter activities of *Drosophila* and shrimp AMPs, such as Drs, Mtk, ALF2, PEN453, and PEN536, after LPS challenge in *Drosophila* S2 cells (56, 59). However, no significant differences have been observed in the mortality rates after *V. alginolyticus, S. aureus*, or WSSV infections in LvFliI-silenced shrimp compared to those of wild-type shrimp (59).

In mammals, SARM is the only negative regulator of the five TLR adaptor proteins—MyD88, TIRAP, TRIF, TRAM, and SARM. By associating with TRIF, SARM functions as an inhibitor of TRIF-dependent signaling (99). No TRIF-dependent signaling has yet been found in invertebrates. In contrast, SARM, such as amphioxus SARM, is able to inhibit the MyD88-dependent signaling pathway by interacting with MyD88 and TRAF6, indicating a negative regulatory role in the Toll pathway (56, 99). One shrimp SARM homolog has been cloned and identified from *L. vannamei*, which is also shown to interact with LvTRAF6, a positive regulatory member of the Toll pathway (58). Knockdown of endogenous LvSARM results in NF-κB activation and enhances the expression levels of NF-κB targeted AMPs such as PENs and ALFs, indicating its negative regulatory role in the shrimp Toll pathway. Unexpectedly, LvSRAM-silenced shrimp are more susceptible to infection by *V. alginolyticus* than that of control shrimp injected with GFP dsRNA, which might be explained LvSARM having additional roles beyond innate immunity, such as maintaining normal growth and development (58).

## The IMD Signaling Pathway in Shrimp

**Figure 4:**
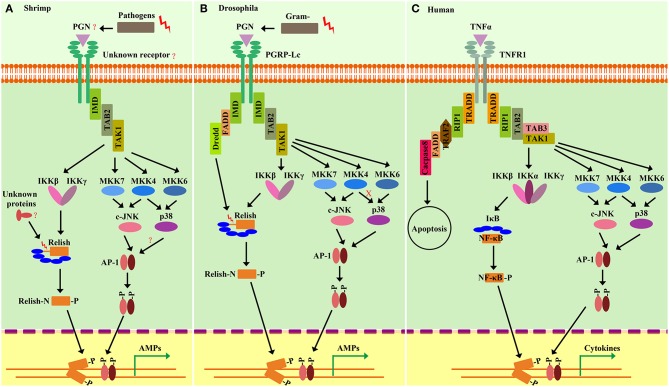
Comparison of the shrimp IMD pathways **(A)** and *Drosophila* IMD pathways **(B)** and *Human* TNFR signaling **(C)**. Homologies between signaling components are depicted by similar shapes and colors. Shrimp and *Drosophila* IMD pathway-signaling events resemble the *Human* tumor necrosis factor receptor (TNFR) signaling pathway. In shrimp, some pivotal components of the IMD pathway, such as the receptors, DREDD, and FADD, are still unknown. Similar to *Drosophila*, pathogen challenges can initiate intracellular signaling events involving IMD, TAB2, and TAK1, which in turn induce the activation of MAPK-AP-1 and IKK-Relish branches. The activation of Relish is required to be phosphorylated by the IKK complex and is cleaved by unknown factors in shrimp. In the MAPK-AP-1 branch of shrimp IMD pathways, the signaling cascade is more similar to that of *Human* than that of *Drosophila*, as manifested by the finding that shrimp p38 can be phosphorylated by MKK4, but *Drosophila* p38 cannot. In shrimp, activation of both of the two branches of IMD pathways can induce the expression of AMPs that confer protection from Gram-negative and Gram-positive bacterial infection.

The IMD pathway was originally defined in *Drosophila* by the identification of a mutation named immune deficiency (IMD) (7568155). The mutated IMD impairs the expression of several AMPs especially Diptericin (Dpt). For this reason, Dpt is often used as a readout of IMD pathway activation, but only marginally affects the Toll-pathway-targeted induction of Drosomycin (Drs) (100, 101). It is generally considered that IMD-deficient flies succumb to Gram-negative bacteria, which is different from that of Toll mutant flies that are more susceptible to fungi and Gram-positive bacteria (21, 100, 102). IMD encodes a death-domain–containing protein similar to the receptor-interacting protein (RIP) of the tumor necrosis factor receptor (TNF-R) pathway in mammals (103). In the TNFR pathway, RIP is essential for both NF-κB and mitogen-activated kinase (MAPK) activation. *Drosophila* IMD mediates a signaling cascade that broadly resembles the mammalian TNFR pathway (104) (Figure 4).

### The Canonical Components of the IMD Pathway

Specifically (Figure 4), the *Drosophila* IMD pathway is trigged by meso-diaminopimelic acid (DAP)-type peptidoglycan (PGN), which comprises the cell wall of most Gram-negative bacteria, as well as some Gram-positive bacteria (100). PGRP-LC and PGRP-LE are the two receptors, which are involved in the IMD pathway with different subcellular location. PGRP-LC is on the plasma membrane while the intracellular PGRP-LE binds specifically to DAP-type PGNs (100, 102). After binding to PGN, these receptors likely dimerize or multimerize and then lead to recruitment of a signaling complex consisting of IMD (101, 105), Fas-Associated protein with a death domain (FADD) (105, 106) and the caspase-8 homolog death-related ced-3/Nedd2-like protein (DREDD) (100, 102, 105, 106). Inhibitor of apoptosis 2 (IAP2), a ubiquitination-machinery component, function as an E3-ubiquitin ligase could activate DREDD via ubiquitination. Activated DREDD cleaves IMD, exposing a binding site for IAP2, leading to K63-ubiquitinate IMD (106). The K63 ubiquitin chain of IMD functions as a scaffold for interaction with TAK1/TAB2 complex. The TAK1/TAB2 complex is responsible for activating both MAPK/AP-1 and IKK/Relish branches of the IMD pathway (105–107). Relish activation is achieved by both IKK-mediated phosphorylation and cleavage by DREDD, after which the N-terminal Rel domain of Relish undergoes nuclear translocation and initiates the transcription of target genes (102, 108).

The studies of IMD pathway in shrimp have just begun in the last 10 years. In 2009, Wang et al. identified an IMD homolog (LvIMD) from *L. vannamei*, which was the first IMD homolog identified in shrimp (17, 64). LvIMD encodes a death domain-containing protein, which is moderately homologous to *Drosophila* IMD and mammalian RIP (17). Studies show that the IMD homologs from different shrimp species are distinctly different in the context of their tissue distributions and their responsiveness to infection. The FcIMD from *F. chinensis* is very abundant in the gills and stomach, whereas the PcIMD of *P. clarkia* exhibits high expression levels in the heart, hepatopancreas, and stomach. In contrast, LvIMD of *L. vannamei* exhibits expression abundantly in the nervous system, gill, intestine, and pyloric caecum (17, 109, 110). Additionally, FcIMD in gills is up-regulated by WSSV but not *Vibrio* challenge, while PcIMD in gills can respond to *Vibrio* but not WSSV (109, 110). LvIMD mRNA can be induced by multiple immune stimuli including LPS, *V. alginolyticus, S. aureus, Saccharomyces cerevisiae* (yeast), and WSSV, both in hepatopancreas and hemocytes but not in gills (17). Since the LvIMD was identified in *L. vannamei*, an increasing number of components of the canonical IMD pathway in shrimp have been identified, including TAK1, TAB1, TAB2, Relish, mitogen-activated protein kinase kinase 3 (MKK3), MKK4, MKK6, MKK7, c-Jun N-terminal kinase (JNK), c-Jun, c-Fos, p38, ATF2, NF-κB repressing factor (NKRF), Akirin, Bap60, and 14-3-3 (12, 17, 73, 105, 109–127). The canonical components of the IMD signaling pathway identified in penaeid shrimp are listed in Table 2.

**Table 2:** Components of canonical IMD signaling pathway identified in shrimps.

**Components**	**Species**	**Gene names**	**Accession numbers**	**In response to pathogenic infections**	**References**
IMD	*L. vannamei*	LvIMD	ACL37048.1	Muscle: *V. anguillarum* and *M. lysodeikticus*, up; Hepatopancreas: *V. alginolyticus*, up; Hemocyte: *V. alginolyticus, S. aureus* and WSSV, up	(17, 24)
	*F. chinensis*	FcIMD	JX867731.1	Cephalothorax: *V. anguillarum* and *M. lysodeikticus*, up; Hemocytes: *V. anguillarum*, down; Hemocytes and gills: WSSV, up	(109, 110)
	*P. clarkii*	PcIMD	Unsubmitted	Hemocytes and gills: *V. anguillarum*, up	(109)
TAK1	*L. vannamei*	LvTAK1	KU522004.1	Gills: *V. parahaemolyticus, S. saureus*, and WSSV, up	(112)
TAB1	*L. vannamei*	LvTAB1	KY683840	Gills: *V. parahaemolyticus* and *S. saureus*, up	(113)
TAB2	*L. vannamei*	LvTAB2	KP780842.1	Gills: *V. parahaemolyticus, S. saureus* and WSSV, up; Hemocytes: *V. parahaemolyticus*, WSSV, up	(105)
IKKβ	*L. vannamei*	LvIKKβ	AEK86518	Gill: *V. alginolyticus* and WSSV, up; Hemocyte: *V. alginolyticus* and WSSV, down; Intestine: *V. alginolyticus* and WSSV, up	(52)
IKKε	*L. vannamei*	LvIKKε1	AEK86519	Gill: *V. alginolyticus* and WSSV, up; Hemocyte: *V. alginolyticus* and WSSV, down; Hepatopancreas: *V. alginolyticus* and WSSV, up; Intestine: *V. alginolyticus* and WSSV, up	(52)
	*L. vannamei*	LvIKKε2	AEK86520	Gill: *V. alginolyticus* and WSSV, up; Hemocyte: *V. alginolyticus* and WSSV, down; Hepatopancreas: *V. alginolyticus* and WSSV, up; Intestine: *V. alginolyticus* and WSSV, up	(52)
Relish	*L. vannamei*	LvRelish	EF432734	Gills: WSSV, up	(12, 12, 53)
	*P. monodon*	PmRelish	KM204120	Hemocytes, lymphoid organ, gill, hepatopancreas and heart: *V. harveyi*, WSSV, YHV, up	(114)
	*F. chinensis*	FcRelish	EU815055.1	Gills: *V. anguillarum*, down	(110).
	*M. rosenbergii*	MrRelish	KR827675.1	Hepatopancreas: *V. anguillarum*, up	(111)
NKRF	*L. vannamei*	LvNKRF	KY864366	Gills: WSSV, up	(117)
Akirin	*L. vannamei*	LvAkirin	KC415269.1	Hepatopancreas: *V. parahaemolyticus*, up	(115)
	*M. japonicus*	MjAkirin	AB503217.1	Hemocytes: *V. anguillarum*, up	(116)
Bap60	*M. japonicus*	MjBap60	KT892952.1	Hemocytes: *V. anguillarum*, up	(116)
14-3-3	*L. vannamei*	Lv14-3-3EL	JF81119	Gill and muscle: WSSV, up; lymphoid organ, WSSV, down	(118)
	*L. vannamei*	Lv14-3-3ES	JF81120	Muscle: WSSV, up Lymphoid organ: WSSV, down	(118)
	*M. japonicus*	Mj14-3-3	KT892951.1	Hemocytes: *V. anguillarum*, down	(116)
JNK	*L. vannamei*	LvJNK	JN035903.1	Gills: WSSV, up	(120)
c-Jun	*L. vannamei*	Lvc-Jun	KM401573.1	Gills: WSSV and *V. parahaemolyticus*, up	(119, 121)
	*P. monodon*	Pmc-Jun	KX216509	Gill and hepatopancreas: *V. harveyi* and *S. agalactiae*, up.	(73)
c-Fos	*L. vannamei*	Lvc-Fos	KP676567	Gills: WSSV and *V. parahaemolyticus*, up	(119)
MKK3	*F. chinensis*	FcMKK3	KF994775	Hemocytes and gills: WSSV, *V. anguillarum* and *S. aureus*, up	(122)
MKK4	*L. vannamei*	LvMKK4	KY693644	Intestine and hepatopancreas: *V. parahaemolyticus, S. aureus* and WSSV, up.	(126)
	*F. chinensis*	FcMKK4	KJ023198	Hemocytes and gills: WSSV, *V. anguillarum* and *S. aureus*, up	(122)
MKK6	*L. vannamei*	LvMKK6	KR535627	Gills: *V. parahaemolyticus, S. aureus* and WSSV, up	(123)
MKK7	*L. vannamei*	LvMKK7	KT719405	Hepatopancreas: *V. parahaemolyticus, S. aureus* and WSSV, up	(125)
P38	*L. vannamei*	LvP38	JN035902.2	Gills and hemocytes: *V. alginolyticus, S. aureus* and WSSV, up	(124, 127)
	*F. chinensis*	FcP38	KF991368	Hemocytes and gills: WSSV, *V. anguillarum* and *S. aureus*, up	(122)
ATF-2	*F. chinensis*	FcATF-2	KF991367	Hemocytes and gills: WSSV, *V. anguillarum* and *S. aureus*, up	(122)
TRIM	*L. vannamei*	LvTRIM9	Unsubmitted	Intestine: WSSV, up	(128)
β-TrCP	*L. vannamei*	Lvβ-TrCP	XM_027360659	ND	(128)

*up, differentially up-regulated; down, differentially down-regulated; ND, Not determined*.

### Cascades of the IMD Pathway

As mentioned above, many members homologous to most of the components of the *Drosophil*a canonical IMD pathway have been cloned and identified in shrimp, and the regulatory network of the shrimp IMD pathway has increasingly become further elucidated (Figure 4). The *Drosophil*a IMD pathway uses the plasma-membrane-located PGRP-LC or the intracellular PGRP-LE to sense microbial DAP-type PGNs, but, until recently, no member of the PGRP family has been found in shrimp (100). We also were unable to find any PGRP homolog by homology searching against various transcriptome data from multiple shrimp species. Nevertheless, it is tempting to speculate that there could be a novel receptor mediating the sensing of microbial infection in shrimp, and that the signaling cascade of the shrimp IMD pathway generally resembles that of the *Drosophil*a IMD pathway (Figure 4).

In particular, a signal-transduction complex consisting of TAK1, TAB1, and TAB2 has been observed in shrimp *L. vannamei*, where TAB2 could be an adaptor to link upstream IMD (105, 112, 113). Similar to the *Drosophil*a IMD pathway, shrimp TAK1 is responsible for activating both the MAPK/AP-1 and IKK/Relish branches of the IMD pathway (102). There are two IKK-related kinases, IKKβ and IKKε, identified in shrimp, but whether they can receive the phosphorylation signaling from TAK1 is still unknown. Moreover, over-expression of shrimp TAK1 has been demonstrated to strongly regulate the promoter activities of the *Drosophila* IMD pathway controlling AMPs, such as Dpt in S2 cells, suggesting a conserved role of shrimp TAK1 in the IKK-Relish branch (112). Additionally, shrimp TAK1, functioning as a mitogen-activated protein kinase kinase kinase (MAPKKK), is able to activate and phosphorylate several MAPKKs, including MKK3, MKK4, MKK6, and MKK7, *in vitro* (unpublished data). Furthermore, MKK7 is recognized as the upstream-kinase target to JNK, while MKK6 is the upstream kinase responsible for p38 (123, 125). Of note, MKK4 from shrimp *L. vannamei* can activate and phosphorylate p38 (126). As to this point of the phosphorylation on p38, the cascade of the IMD pathway in shrimp is more similar to mammals than that of *Drosophila*. In shrimp, p38 can be activated and phosphorylated by both MKK4 and MKK6 (123, 126). Additionally, shrimp TAB1 is able to combine with p38, and, thus, it could be an important regulatory subunit for p38 (113). However, in *Drosophil*a, there is no TAB1 homolog, and it has been definitively shown that MKK4 does not active p38 (129). So far, the activation of p38 in shrimp includes at least three routes: the MKK4-p38, MKK7-p38, and TAB1-p38 pathways. Such multiple pathways might supply a fine-tuning control of p38 activity to pathogenic invasion. It also suggests an important role of p38 in shrimp immunity. For example, when one or two routes are blocked under some specific condition, p38 can still work in the immune response.

c-Fos and c-Jun, belonging to the activator protein-1 (AP-1) family, are the transcription factors of the IMD-MAPK branch (130). Shrimp c-Jun has been observed to be mainly located in the nucleus of insect cells under non-stimulated signaling conditions. So, it is inferred that under stressed, immune conditions, JNK could translocate from the cytoplasm to the nucleus where it could phosphorylate and activate c-Jun, which in turn could induce the expression of JNK-pathway-target genes. This hypothesis is supported by the observation that JNK-phosphorylation levels are evidently reduced by JNK-inhibitor (SP600125) treatment *in vivo* (120, 121). More direct evidence should be demonstrated to further explore whether JNK can interact with and phosphorylate c-Jun *in vitro* (119). Next, c-Fos, another member of the AP-1 family, has been shown to form a heterodimer with c-Jun, which is also able to form a homodimer with itself, suggesting that c-Fos might also be downstream substrate of JNK (119). The ATF2, a member of the ATF/cAMP response-element-binding family of transcription factors, contains a common feature of the bZIP element, which is able to form homodimers or heterodimers with other proteins that contain bZIP elements, such as the AP-1 (131). A shrimp ATF2 homolog, FcATF2, has been reported in *F. chinensis*. Silencing of Fcp38 results in a reduction in the transcription of FcMKK3 and FcATF2, indicating that shrimp MKK3, p38, and ATF2 might function in the same signaling route (122). However, whether FcMKK3 can directly regulate the phosphorylation of Fcp38, and whether Fcp38 has the ability to activate FcATF2, need to be determined in further investigations.

Relish, another member of the NF-κB family, is the transcription factor of the IKK-Relish branch, both in *Drosophila* and shrimp. Similar to *Drosophila* Relish, shrimp Relish proteins are master regulators for the synthesis of a wide range of AMPs. Multiple Relish homologs have been identified from various shrimp species. The full length of LvRelish from *L. vannamei* consists of an N-terminal Rel homology domain (RHD), a nucleus-localization signal (NLS), and an IκB-like domain containing six ankyrin repeats (ANKs) and a death domain (DD) in the C-terminal. A truncated isoform of Relish, sLvRelish, has also been found in *L. vannamei* and it shares the RHD region with LvRelish but does not have several domains, including NLS, ANKs and DD (12). LvRelish can bind to a κB-response element from *Drosophila* and regulate the transcription of several AMPs such PEN2, PEN4, and Atta (12, 53). The PmRelish from *P. monodon* has been shown to regulate the synthesis of AMPs such as PEN5, PEN3, ALFPm3, and ALFPm6 in response to *V. harveyi* or yellow head virus (YHV) infection (132, 133). In *M. rosenbergii*, Shi et al. show that over-expression of MrRelish in S2 cells induce the expression of both *Drosophila* and shrimp AMPs, such as *Drosophila* Mtk, Atta, Drs, and Cecropin A (CecA) and shrimp PEN4 (111). Furthermore, RNAi of MrRelish leads to reduced expression of Crustin (Cru) 2, Cru5, Cru8, Lysozyme (Lyz) 1, and Lyz2, but not ALF1 and ALF3, *in vivo* (111). Wang et al. used the combined methods of RNA interference (RNAi) and suppression-subtractive hybridization (SSH) to screen *F. chinensis* Relish-regulated genes, and a large amount of genes were identified and could be involved in multiple biological processes, such as immunity, development, metabolism, and genetic-information processing (134). This study has provided a novel view to understand the function of Relish beyond its conventional role in regulating AMPs in innate immunity.

### Regulators of the IMD Pathway

Recent reports have identified several regulators of the IMD-Relish pathway in shrimp, such as NF-κB repressing factor (NKRF), Akirin, and TRIpartite Motif 9 (TRIM9) (115, 116, 128). In mammals, NF-κB repressing factor (NKRF) is well-recognized as a suppression factor for NF-κB, which specifically counteracts the basal activity of several NF-κB-dependent promoters by binding directly to specific negative-regulatory DNA elements (NRE) (135). In contrast, *L. vannamei* NKRF shows no inhibitory effects but instead exhibits enhancing effects on activities of Dorsal and Relish, as observed by the fact that NKRF can directly interact with both of the NF-κB members to regulate the promoter activities of PEN4, a previously identified target gene of Toll and IMD pathways (117). Shrimp Akirin homologs are recently discovered nuclear factors that play important roles in innate immune system. Two Akirins have been identified in *L. vannamei* and *M. japonicas*, respectively (115, 116). By RNAi methods, both LvAkirin and MjAkirin have been shown to positively regulate the expression of several IMD-Relish-target AMPs *in vivo* (115, 117). MjAkirin is able to regulate IMD-Relish-target AMPs, which could be attributed to its direct interaction with Relish. Interestingly, MjAkirin could function as an important regulator for Bap60 and 14-3-3 to positively and negatively regulate the activity of the IMD-Relish pathway, respectively (116). As a bridge protein, MjAkirin links the transcription factors, Relish and Bap60, the latter of which is a component of the Brahma (SWI/SNF) ATP-dependent chromatin-remodeling complex and positively regulates AMP expression (115, 116). On the other side, the heterotrimeric complex, comprised of Akirin, Relish, and 14-3-3, has been shown to down-regulate AMP expression by an unknown mechanism (116, 118). Recently, an E3-ubiquitin ligase, TRIM9, has been identified in shrimp *L. vannamei*. LvTRIM9 can directly interact with beta-transducin repeat-containing protein (β-TrCP), an inhibitor of the NF-κB pathway, and down-regulate the expression levels of LvRelish and AMPs, which suggests that WSSV may hijack host LvTRIM9 for its propagation through inhibition of the NF-κB pathway and AMP production via the interaction of LvTRIM9 with Lvβ-TrCP (128). These observations suggest that the IKK-Relish branch may be under multiple layers of control and may crosstalk with many other pathways because 14-3-3 is conserved protein that is implicated with a wide variety of signal-transduction pathways.

## NF-κB (Toll and IMD) Pathways Regulate AMPs Expression in Response to Bacterial Infection

Dorsal and Relish are the downstream NF-κB-family transcription factors of Toll and IMD pathways, respectively. In *Drosophila*, the Toll pathway responds to Gram-positive bacteria and fungi, while the IMD pathway responds to the Gram-negative pathway (20). However, in shrimp, it is very interesting to find that there is no specific response to Gram-positive or Gram-negative bacteria by Toll and IMD pathways, as demonstrated by the findings that many pivotal components from the shrimp Toll pathway—such as Toll, MyD88, and Dorsal—and from the IMD pathway—such as IMD, TAK1, and Relish—are activated in response to both Gram-negative bacteria and Gram-positive bacteria (12, 16, 17, 24, 25, 28, 30, 31, 35, 36, 39, 105, 110–113). Similar to the *Drosophila* NF-κB pathway, the activation of shrimp NF-κB pathways leads to the boosted expression of various AMPs, which are widely considered to be the major antimicrobial effectors in humoral immunity. AMPs are an group of molecules with molecular weights that are usually <10 kDa, which are effective on bacteria (Gram-positive and Gram-negative), fungi (yeasts and filamentous), and parasites, as well as in some cases on enveloped viruses (136). AMPs are found in evolutionarily diverse organisms ranging from prokaryotes, invertebrates, vertebrates, and to plants (137–139). To date, several classes of AMPs or effectors have been identified in shrimp, composed of Penaeidin (PEN), Crustin (Cru), anti-LPS-factor (ALF), C-type lectin (CTL), Lysozyme (Lyz), and thioester-containing protein (TEP). In *Drosophila*, AMPs are mainly regulated by NF-κB pathways, and the transcription of drosomycin (Drs) and diptericin (Dpt) have been identified as the hallmarks for the activation of the Toll pathway and the IMD pathway, respectively (22, 23, 136). However, the regulatory mechanism of AMPs is still not clear in shrimp. Herein, we will summarize these AMPs and other antimicrobial proteins that have been reported to be associated with the two NF-κB pathways, directly or indirectly (Table 3).

**Table 3:** AMPs or effectors related to NF-κB pathways identified in shrimps.

**AMPs**	**Species**	**Types**	**Function identified**	**Signaling pathways**	**References**
LvPEN2	*L. vannamei*	Penaeidin	Anti-*Vibrio*	AP-1 (c-Fos, c-Jun)	(27, 119, 140)
LvPEN3	*L. vannamei*	Penaeidin	Anti-*Vibrio*	Toll, AP-1	(24, 27, 119, 140)
LvPEN4	*L. vannamei*	Penaeidin	Anti-*Vibrio*	IMD; Toll2; NF-κB; AP-1	(11, 12, 17, 27, 119, 140, 141)
LvCru1	*L. vannamei*	Crustin	*V. alginolyticus*: down	Toll4; TAK1; IAP	(27, 112, 142, 143)
LvCru2	*L. vannamei*	Crustin	ND	TAK1	(112)
LvCru3	*L. vannamei*	Crustin	ND	Toll4; TAK1; IAP	(27, 112, 143)
LvCrustinA	*L. vannamei*	Crustin	Anti-*Vibrio*; Anti-WSSV	NF-κB; AP-1	(141)
LvCrustinP	*L. vannamei*	Crustin	ND	Toll; IMD; AP-1; NF-κB	(24, 144)
LvALF1	*L. vannamei*	Anti-LPS-factor	Interact with VP19, VP26, VP28, wsv134, and wsv321	IMD; Toll4; Dorsal	(24, 27)
LvALF2	*L. vannamei*	Anti-LPS-factor	Anti-*Vibrio*; Anti-fungi; Anti-WSSV	Toll4; Dorsal	(27, 145)
LvALF3	*L. vannamei*	Anti-LPS-factor	Anti-*Vibrio*; anti-WSSV	Toll4; Dorsal	(27)
LvCTL3	*L. vannamei*	C-type lectin	Anti-*Vibrio*; anti-WSSV	Dorsal	(146)
LvCTL4	*L. vannamei*	C-type lectin	Anti-*Vibrio*	NF-κB	(147)
LvLYZ1	*L. vannamei*	Lysozyme	Anti-*Vibrio*; Anti-WSSV: Interact with VP26, VP28, wsv134, and wsv321	Toll4; Dorsal; TAK1	(27, 112)
LvLYZ2	*L. vannamei*	Lysozyme	Anti-*Vibrio*; Anti-WSSV	Toll4; Dorsal; TAK1	(27, 112)
LvLYZ3	*L. vannamei*	Lysozyme	Anti-*Vibrio*; Anti-WSSV	Dorsal	(27)
LvLYZ4	*L. vannamei*	Lysozyme	ND	Toll4; Dorsal; TAK1	(27, 112)
LvTEP1	*L. vannamei*	TEP	G+/G-: up; WSSV: up	NF-κB; AP-1	(148)
FcPEN3	*F. chinensis*	Penaeidin	ND	Relish	(149, 150)
FcPEN5	*F. chinensis*	Penaeidin	Anti-bacteria	NF-κB	(13, 14, 151)
FcCru1	*F. chinensis*	Crustin	ND	IMD; Relish	(109, 149, 152)
FcCru2	*F. chinensis*	Crustin	ND	Spz	(41, 152)
FcCru3	*F. chinensis*	Crustin	ND	IMD	(109, 152)
FcALF	*F. chinensis*	Anti-LPS-factor	ND	Relish	(149)
FcALF6	*F. chinensis*	Anti-LPS-factor	ND	IMD	(109, 153)
FcALF8	*F. chinensis*	Anti-LPS-factor	ND	IMD	(109)
FcLys2	*F. chinensis*	Lysozyme	ND	IMD	(109)
PmPEN3	*P. monodon*	Penaeidin	Anti-bacteria	Spz	(39, 154)
PmPEN411	*P. monodon*	Penaeidin	ND	AP-1, Dorsal	(155)
PmPEN536	*P. monodon*	Penaeidin	ND	AP-1, Dorsal	(119, 155)
PmPEN309	*P. monodon*	Penaeidin	ND	Toll2	(26)
Crus-likePm	*P. monodon*	Crustin	*V. harveyi*: up	NF-κB; STAT5; AP-1	(26, 156)
crustinPm1	*P. monodon*	Anti-LPS-factor	ND	Spz	(39)
crustinPm5	*P. monodon*	Anti-LPS-factor	ND	NF-κB	(26, 157)
crustinPm7	*P. monodon*	Anti-LPS-factor	ND	Spz	(39)
ALFPm2	*P. monodon*	Anti-LPS-factor	*V. harveyi*: up	NF-κB; AP-1	(158, 159)
ALFPm3	*P. monodon*	Anti-LPS-factor	*V. harveyi*: up; WSSV: up	Spz	(39, 153, 158, 159)
MjLys1	*M. japonicas*	Lysozyme	*V. anguillarum*: up;	Toll; IMD; Toll3	(109, 160, 161)
Mj-Lys2	*M. japonicus*	Lysozyme	ND	IMD	(109)
PcCru1	*P. clarkia*	Crustin	*V. anguillarum*: up	Toll; IMD; Toll3	(19, 36, 109)
PcCru2	*P. clarkia*	Crustin	*V. anguillarum*: up	Toll; IMD	(19, 109)
PcALF1	*P. clarkii*	Anti-LPS-factor	anti-bacteria	IMD; Toll4; Toll2; Toll3	(35–37, 109, 162)
PcALF2	*P. clarkia*	Anti-LPS-factor	*V. anguillarum*: up	IMD; Toll; Toll4; Toll2	(19, 35, 37, 109)
PcALF4	*P. clarkia*	Anti-LPS-factor	ND	Toll4	(37)
PcALF7	*P. clarkia*	Anti-LPS-factor	ND	Toll4	(37)
PcALF10	*P. clarkia*	Anti-LPS-factor	ND	Toll4	(37)
PcLys1	*P. clarkii*	Lysozyme	*V. anguillarum*: up;	Toll; IMD; Toll3	(19, 36, 109)
MrCru2	*M. rosenbergii*	Crustin	Anti-WSSV	Relish	(111)
MrCru3	*M. rosenbergii*	Crustin	Anti-WSSV	Toll	(34)
MrCru5	*M. rosenbergii*	Crustin	Anti-WSSV	Relish	(111)
MrCru7	*M. rosenbergii*	Crustin	Anti-WSSV	Toll	(34)
MrCru8	*M. rosenbergii*	Crustin	Anti-WSSV	Relish	(111)
MrALF2	*M. rosenbergii*	Anti-LPS-factor	ND	Toll	(34)
MrALF3	*M. rosenbergii*	Anti-LPS-factor	ND	Toll	(34)
MrALF4	*M. rosenbergii*	Anti-LPS-factor	ND	Toll	(34)
MrALF5	*M. rosenbergii*	Anti-LPS-factor	ND	Toll	(34)
MrLys1	*M. rosenbergii*	Lysozyme	ND	Relish	(111)
MrLys2	*M. rosenbergii*	Lysozyme	ND	Relish	(111)

*G^+^, Gram-positive bacteria; G^−^: Gram-negative bacteria; up, differentially up-regulated; down, differentially down-regulated; ND, Not determined*.

PENs are a special class of AMPs that have only been identified in Penaeid shrimp. Delphine et al. isolated three PENs from the hemolymph of shrimp *P. vannamei* for the first time in 1997 (163). These peptides are defined as the name of Penaeidins after the genus Penaeus, which therefore cannot be associated to groups hitherto described (163). This family is highly cationic, consisting of a highly conserved leader peptide followed by an N-terminal proline-rich domain (PRD) and a C-terminal cysteine-rich domain (CRD). PENs have been shown to possess strong antimicrobial activities against Gram-positive and Gram-negative bacteria, as well as fungi (164). Until recently, PENs had only been discovered in several shrimp, including *L. vannamei, F. chinensis, P. monodon*, and *M. japonicus*. Unlike LvPEN2 and LvPEN3, the LvPEN4 upstream-regulatory region contains many putative transcription-factor-binding sites, including STATx, AP-1, Dorsal, and GATA (140). Luciferase-reporter assays have confirmed that LvToll2, LvToll4, LvMyD88, LvDorsal, LvIMD, and LvRelish, which belong to either IMD or Toll pathways, are able to induce the promoter activity of LvPEN4 in insect cells (11, 12, 17, 26, 27). Chromatin immunoprecipitation (CHIP) assays have also demonstrated that LvDorsal can bind with the promoter region of LvPEN4 after LPS challenge *in vivo*, which correlates well with the finding that over-expression of LvCactus represses the promoter activity of LvPEN4 *in vitro* (55). Moreover, LvPEN2, LvPEN3, and LvPEN4 have been shown to be up-regulated by IKKβ, TAK1, MKK4, MKK6, and AP-1 (c-Fos and c-Jun) *in vivo* or *in vitro*, which are key components of the IMD pathway (52, 112, 119, 123, 126). However, Hou et al. observed that knockdown of IMD only induced subtle effects on the expression of PEN3 *in vivo* after *V. anguillarum* and *M. lysodeikticus* infections (24). The discrepancy of these results may be due to differences between the evaluated methods on expression of PEN3 and/or different bacterial challenges used in the studies. In *F. chinensis*, knockdown of FcRelish results in down-regulation of FcPEN3 after bacterial infection *in vivo* (149). FcPEN5, with strong activities against Gram-positive and Gram-negative bacteria as well as fungi, has been shown to be greatly suppressed and delayed in Relish-silenced shrimp after *Vibrio anguillarium* and *Micrococcus lysodeikticus* challenges (13, 14, 151). Additionally, Ho et al. cloned the following two types of genomic fragments flanking the 5′ end of the Penaeidin gene in *P. monodon*: Type536 and Type411 sequences, both of which contain several transcription-factor-binding motifs, such as TATA box, GATA, dorsal, and AP-1 (155). Similar to PEN2-4 from *L. vanamei*, many members from either IMD or Toll pathways can induce the promoter activities of Type536 and Type411 in insect cells (119, 155). In summary, PENs have been found in several penaeid shrimp and have broad anti-microbial properties to Gram-positive and Gram-negative bacteria, as well as fungi (151, 164), but they do not exhibit any antiviral properties. Most PENs can be regulated by both the Toll pathway and two branches of the IMD pathway.

Crustins, functioning as protease inhibition or as an effector molecule, is a type of whey acidic protein (WAP) domain-containing and cationic cysteine-rich AMPs (141). Generally, the characterized WAP domain is composed of 50 amino-acid residues with eight cysteine residues at defined positions, which form four intracellular disulfide bonds to create a tightly packed structure (141, 144). Crustins are usually classified into five types (Type I–V) based on the differences in the domain organizations between the signal sequences and the WAP domain (165). Most Crustins found in shrimp belong to Type-II Crustins that contain an N-terminal signaling peptide, followed by a long glycine-rich domain, cysteine-rich domain, and a WAP domain at the C-terminal. There are also some Crustins in shrimp belonging to Type III, which are also named single-WAP-domain proteins (SWDs) (26, 144). Instead of the glycine-rich and the cysteine-rich regions, SWDs have a proline-arginine-rich domain between the signal sequence and the WAP domain (144). In shrimp, most Crustins are mainly expressed in the hemocytes or gills, but rarely in hepatopancreas (141, 144, 152, 166, 167). Surprisingly, PmCru5 from *P. monodon* could not be detectable in hemocytes but was highly expressed in the epipodite and eyestalks (157). The transcription of Crustins in shrimp might be regulated by both Toll and IMD pathways and other unknown signaling pathways. Analyzing 5′-upstream sequence of Crustins reveal that there are a putative TATA box and several putative binding sites for NF-κB, AP-1, and STAT5 (24, 26, 34, 36, 109, 141, 144, 149). Interestingly, in addition to the above potential binding sites, the promoter of PmCru5 also contains a complete heat-shock-regulatory element, indicating that heat shock may also induce the expression of PmCru5 (144, 157). Studies from Wang et al. have showed that silencing of LvIAP2, homologous to *Drsosophila* IAP2 of the IMD pathway, leads to a decrease in the expression of LvCrustin1 and LvCrustin3 in hemocytes (143). Zhang et al. have reported that the expression of a Crustin from *L. vannamei* is up-regulated when Flightless-I is silenced (168). Recently, Li et al. have reported a new Crustin from *L. vannamei*, named LvCrustinA, which is abundantly expressed in immune-related tissues, such as the gill, hemocyte, and epithelium (141). Dual-reporter assay in S2 cells shows that LvCrustinA can be induced by LvDorsal, LvRelish, and Lvc-Jun, suggesting that LvCrustinA could be regulated by both IMD and Toll pathways (141). Lan et al. have shown that the IMD pathway participated in inducing of three kinds of AMP genes, namely, Crustins, ALFs, and Lysozymes, in *F. chinensis* and *P. clarkii*. Specifically, Cru1, Cru2, ALF1, ALF2, and Lyz1 in *P. clarkii*, and Cru1, Cru3, ALF6, ALF8, and Lyz2 in *F. chinensis* are thought to be induced via the IMD pathway after *V. anguillarum* challenge (109). Feng et al. have reported that two Crustins, Cru3 and Cru7, from *M. rosenbergii* are down-regulated in gills of MrToll-knockdown shrimp in response to WSSV infection (34). However, a Crustin from *L. vannamei* has been shown to not be regulated by Toll or IMD genes *in vivo* (24). Most of the Crustins identified from shrimp have been reported to have antiviral or antibacterial roles (169–174), but their expressions implicated in signaling pathways are still unclear. For example, MjCru from *M. japonicus* has been shown to possess antibacterial activity against Gram-positive and Gram-negative bacteria through destroying the surface of bacterial cells (141, 172). Recently, two new Crustin isoforms, MjCRS8 and MjCRS9, from *M. japonicus* have been shown to only be expressed in gills, but they do not respond to *V. parahaemolyticus* or WSSV by immersion tests, which suggests that some Crustins could have additional roles beyond immunity (175).

ALFs are a group of AMPs that were firstly isolated from the hemocytes of the horseshoe crabs, *Limulus polyphemus* (144, 176). *L. polyphemus* ALF binds LPS, inhibited the LPS-mediated activation of the *Limulus* coagulation system, and could strongly inhibited the growth of Gram-negative bacteria (177). In shrimp, ALFs were first identified from the hemocytes of *P. monodon* (158). Antimicrobial *in vitro* assays performed with recombinant PmALFs have shown a strong activity against Gram-positive and Gram-negative bacteria and filamentous fungi (178). Subsequently, *in vivo* experiments, LvALF1-knockdown shrimps exhibited more sensitive to both bacterial and fungal infection than the control group (145). Recently, a new ALF isoform (LvALF AV-R) has been shown to have higher expression levels in hepatopancreas of VP_PirA/B-like toxin-resistant shrimp. The recombinant LvALF AV-R has been found to bind with bacterial proteins, but not Vp_PirAB-like toxin, which suggests that LvALF AV-R might be involved in the resistance mechanism in a non-direct manner (179). So far, several ALFs from several shrimp species—such as *L. vannamei* and *F. chinensis*—have been reported to be regulated by the IMD pathway, as indicated by the decreased expression of ALFs in IMD- or Relish-knockdown shrimp (24, 109, 149). Huang et al. have shown that 5 of 11 ALFs (PcALF1, PcALF2, PcALF4, PcALF7, and PcALF10) from *P. charkii* are regulated by PcToll4 after WSSV challenge in the intestine, but the expressions of other ALFs do not change significantly when PcToll4 is silenced (37). Interestingly, Lan et al. have reported that PcToll2 from *P. charkii* can positively regulate the expression of PcALF1 and PcALF2 through activating the transcription factor PcATF4, but not PcDorsal or PcSTAT, to defend against Gram-negative bacteria (35). Additionally, as mentioned earlier, Lan et al. also reported that several ALFs from *F. chinensis* and *P. clarkia* can be regulated by the IMD pathway to defend against bacterial infection (109). Feng et al. have determined that the MrToll from *M. rosenbergii* can regulate the expression of four ALF genes (MrALF2, MrALF3, MrALF4, and MrALF5 genes) in the gills after WSSV infection (34). ALFs have also been discovered in hydrothermal-vent shrimp *Rimicaris* sp. and exhibit activities against a wide range of bacteria (180). In a recent study, the analysis of the tissue distribution, regulation, and biological functions of ALF genes in shrimp suggest that functional diversification of ALFs may rely on multiple selection pressures (159). In addition to the Toll and IMD pathways, other signaling pathways, such as the JAK-STAT pathway, have been shown to participate in regulating the expression of ALFs and Crustins (181).

In crustaceans, non-self-recognition molecules, lectins, play a major role in immune responses mainly by inducing phagocytosis against bacterial pathogens through opsonization (182). C-type lectins (CTLs) are one of the lectin families and is widely existed in Metazoa. C-type lectin domain (CTLD) is the characteristic domain of CTLs, including two disulfide bridges composed of four conserved cysteine residues. Several kinds of CTLs have been identified in shrimp in recent years and have been well reviewed in a previous paper (160). Some CTLs could function in an AMP-like manner, such as FcLec1 and LvCTL3, which could agglutinate both Gram-positive and Gram-negative bacteria (160, 183). There are also reports that have shown that CTLs are regulated by the NF-κB pathway in *L. vannamei*. A NF-κB-binding site has been found in the LvCTL3 promoter and over-expression of LvDorsal can significantly induce the expression of LvCTL3, which came from the first report on the signaling pathway involved in shrimp CTL expression (146). Subsequently, LvCTL4 has also been found to be regulated by both of the two NF-κB proteins, LvDorsal and LvRelish (147). Considering that the expression of most lectins can been rapidly altered in response to diverse pathogens (160), the transcription of lectins induced by host-signaling pathways could be a generally important immune mechanism in shrimp.

Lysozyme (Lyz/ Lys) is known to be an important immune effector, especially for aquatic animals, in resisting bacterial pathogens by lysing bacterial cell walls. Lysozyme has already been identified in several shrimp species and the transcription levels of Lysozymes vary strikingly after bacterial and viral challenges. Only a few studies have reported that shrimp IMD pathway genes, such as IMD and IAP2, have the ability to regulate Lysozymes, including LvLys, FcLys2, Mj-Lys1, Mj-Lys2, and PcLys1 (109, 143, 160, 184). Additionally, PcLysi1 from *P. clarkii* possesses antimicrobial activity and has been shown to be regulated by Toll and Toll3 (184). Although many reports have indicated that shrimp Lysozymes have a broad spectrum of antimicrobial properties against multiple bacteria and viruses (19, 185–194), the information on their transcriptional regulation is still very limited.

In invertebrates, TEPs have been studied deeply in *Anopheles gambiae* (195–198). AgTEP1 has been reported to promote the uptake of bacteria and fungi (199, 200). The first TEP member that was found in crustaceans is *Pacofastacus leniusculus* TEP, which exclusively is expressed in cuticular tissues, such as the gill and intestine (201). In recent years another TEP has been reported in *L. vannamei*. Bacterial treatments degrade the full-length LvTEP1 into a processed fragment, which can bind to both Gram-negative and Gram-positive bacteria (148). Knockdown of LvTEP1 *in vivo* increases the susceptibility to both Gram-positive and Gram-negative bacteria, as well as WSSV. Additionally, the expression of LvTEP1 is dependent on two NF-κB factors, LvRelish and LvDorsal, via the sole NF-κB-binding motif and the AP-1 factors, Lvc-Jun and Lvc-Fos, via the AP-1 motifs in the LvTEP1 promoter (148). Therefore, the authors of this particular study proposed that TEP1 could be induced by both the Toll and IMD pathways (148).

## Two NF-κB Pathways in Response to WSSV Infection

The function of shrimp NF-κB pathways is still elusive in response to viral infection, especially WSSV. WSSV is a large (80–120 × 250–380 nm), non-occluded, rod-shaped, enveloped, and double-stranded DNA virus with a genome of ~300 kbp. WSSV is highly pathogenic and virulent, especially in penaeid shrimp, and it has caused serious yearly economic damage to the shrimp industry worldwide. Shrimp with acute infection by WSSV generally begin to die after 24 h with cumulative mortality of 100% within 3–10 days. WSSV is the most studied viral pathogen in shrimp, which provides a promising possibility to elucidate the interplay between NF-κB pathways and this virus.

**Figure 5:**
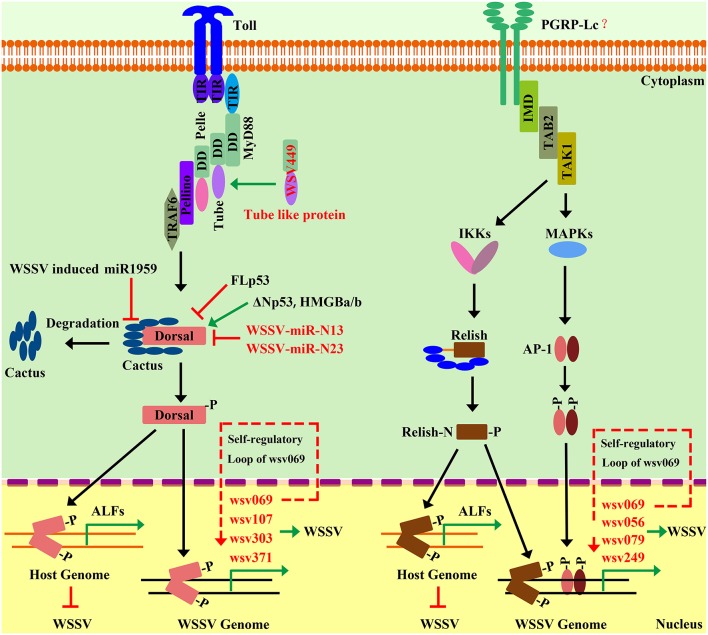
Interplay between shrimp NF-κB pathways and WSSV. The activation of the NF-κB pathway can be hijacked by WSSV to favor its gene expression and genome replication. WSSV infection activates the host Toll pathway, which leads to the activation of Dorsal that translocates into the nucleus to induce viral-gene expression and promote viral-genome replication. A similar situation is observed for the IMD pathway. The activation of wsv069 mediated by the NF-κB pathways can in turn induce its own expression, which creates a self-regulatory loop. Such a positive-feedback loop amplifies the signaling extent to further activate other viral genes. Until now, after WSSV infection, the NF-κB pathways have been shown to be subject to numerous regulatory controls by both host factors—such as p53, miR1959, and HMGB—and viral factor, such as WSSV449, WSSV-miR-N13, and WSSV-miR-N23. However, the activation of the shrimp canonical NF-κB pathway can also lead to AMP expression, such as ALFs, which have strong antiviral activity against WSSV. Therefore, WSSV could have evolved some currently unknown strategies to attenuate the antiviral role of the host NF-κB pathway to instead engage its activation to favor viral pathogenesis.

As mentioned above, the activation of the two shrimp NF-κB pathways (Toll and IMD pathways) leads to the expression of AMPs and other effectors, which confer resistance to a wide range of bacterial pathogens. It is noteworthy that some of these NF-κB pathway-controlled proteins, such C-type lectins and ALFs, possess direct antiviral activities. For instance, the Toll-Dorsal pathway controlling of several AMPs—such as PENs, ALFs, and LYZs—have been shown to interact with different structural proteins of WSSV (27). In addition, knockdown of LvCTL3, a Toll-Dorsal pathway-controlled C-type lectin, has been shown to render shrimp more susceptible to WSSV (146). There are many reported C-type lectins in shrimp with antiviral activity against WSSV—such as LvCTL1, FcLec3, MjLecA, MjLecB, and MjLecC—through their direct interactions with WSSV-envelope proteins; however, whether their expressions are regulated by NF-κB pathways requires further investigation (160, 161, 202, 203). Another case is LvTEP1, which is well identified to be regulated by several transcription factors, including LvDorsal, LvRelish, Lvc-Jun, and Lvc-Fos, which are downstream transcription factors of Toll and IMD pathways. Higher mortality has been observed in LvTEP1-silenced shrimp after WSSV infection, indicating its important antiviral role (63, 148). Additionally, shrimp ALFs have been extensively studied for their roles during WSSV infection. An increasing number of reports have shown that the NF-κB-pathway-targeted ALFs play important role to defend against WSSV. PcToll4 from *P. clarkia* is able to regulate the expressions of five PcALFs in the intestine to oppose WSSV (37). Moreover, five ALFs from *M. rosenbergii* have been shown to be regulated by MrToll to confer protection from WSSV (34). Mechanistically, shrimp ALFs exhibit their antiviral activity via interacting with WSSV structural proteins, therefore interfering with viral invasion (27). In accordance with this potential mechanism, PlALF has been shown to play important roles in protect shrimp from WSSV infection via interfering with viral replication *in vitro* and *in vivo* in crayfish *P. leniusculus* (204), while in red-claw crayfish *C. quadricarinatus*, CqALF can disrupt WSSV-envelope integrity that leads to a decrease of WSSV infectivity (205). PmALF3 from *P. monodon* has been shown to exhibit the ability to interact with several WSSV structural proteins, such as wsv131 (WSSV186), wsv134 (WSSV189), and wsv339 (WSSV395) (206). Therefore, we propose that the NF-κB pathway regulating AMPs to resist WSSV could be a conserved action in different shrimp species (Figure 5). Additionally, WSSV has been shown to have evolved some strategies to inhibit host NF-κB signaling. This strategy of WSSVs is executed via encoded microRNAs. Ren et al. have identified two viral microRNAs (WSSV-miR-N13 and WSSV-miR-N23), which can target Dorsal to suppress the Spz-Toll-Dorsal-ALF antiviral-signaling pathway in shrimp *M. japonicus in vivo* (42, 207).

There are more and more studies showing that the activation of the NF-κB pathway could be hijacked by WSSVs to favor its own gene expression and genome replication (Table 4). The Toll-Dorsal pathway has been shown to be regulated by WSSV at multiple levels, especially via the Dorsal/Cactus complex (Figure 5). Until now, it has still been unclear as to how the host senses WSSV infection and activates the NF-κB pathway. WSSV encodes a WSSV449 protein that is homologous to the host, Tube, which is an adaptor of the MyD88-Tube-Pelle heterotrimeric complex in the Toll pathway. Similar to the host, Dorsal, WSSV449 is able to activate promoters of Toll-pathway-controlled AMPs, as well as the three viral genes, wsv069, wsv303, and wsv371, in insect cells. Therefore, the authors suspected that WSSV449 could activate the Toll-Dorsal pathway for regulating viral gene expression (47). At the layer of Cactus, WSSV infection can induce host miR-1959 in order to reduce the NF-κB inhibitor LvCactus level, thereby leading to the activation of LvDorsal, which in turn translocates into the nucleus to promote viral-gene expression and promote viral-genome replication (94). Additionally, several host factors—such as two p53 isoforms (LvΔNp53 and LvFLp53), two HMGB isoforms (LvHMGBa and LvHMGBb), and LvSTAT from *L. vannamei*—have been shown to regulate viral-gene expression through interacting with LvDorsal (121, 208, 209). In accordance with this, several studies have reported that the promoters of some WSSV genes contain binding sites for NF-κB (12, 42, 63, 117, 209). LvDorsal has been demonstrated to regulate the promoter activities of many viral genes, including wsv051, wsv056, wsv069, wsv078, wsv079, wsv080, wsv083, wsv091, wsv094, wsv098, wsv100, wsv101, wsv103, wsv108, wsv178, wsv249, wsv303, wsv358, wsv371, wsv403, and wsv465 (42, 109, 208). A similar situation exists in the IMD pathway (Figure 5). The two branches of the IMD pathway can be activated by WSSV infection in an unknown manner, and the activated transcription factors, LvRelish, Lvc-Jun and Lvc-Fos, have been shown to regulate a bulk of viral genes, such as wsv056, wsv069, wsv079, and wsv249 (52, 119, 123, 210, 211). Specifically, wsv069 was the first identified immediate-early gene in WSSV, is the most studied viral gene regulated by many host factors, and thus may be a promising case to investigate its regulation via signaling pathways. The promoter-regulatory region of wsv069 contains several binding motifs, including one NF-κB, one STAT, and two AP-1s (210). Until now, host transcription factors—Dorsal, Relish, STAT, YY1, c-Jun, and c-Fos—have all been shown to be involved in the regulation of wsv069 (119, 210, 212, 213); this suggests that after WSSV infection, the expression of wsv069 could be induced by the activation of multiple host-signaling pathways, such as the Toll-Dorsal pathway, the two branches of the IMD pathway, and the JAK-STAT pathway. Of note, the activation of wsv069 by host factors can in turn induce viral genes such as itself, which thus establishes a positive-feedback loop (210). Such positive-feedback loops could amplify the signaling extent to further activate other viral early and late genes. Based on these data, we hypothesize that WSSV may hijack host NF-κB pathways to achieve successfully infection. Some *in vivo* evidence supports this hypothesis, as shown by the fact that knockdown of many pivotal components of NF-κB pathways—such as Dorsal, Relish, and AP-1—have lower viral loads and render shrimp more resistant to viral infection (11, 53, 73, 114, 119).

**Table 4:** WSSV proteins/genes related to host (shrimps) Toll and IMD pathways—proteins/genes.

**WSSV genes/proteins**	**Molecular interaction with host Toll and IMD pathways genes/proteins**	**Species**	**References**
wsv390/WSSV449	Tube-like protein	*L. vannamei*	(47)
WSSV-miR-N13, WSSV-miR-N23	LvDorsal	*M. japonicus*	(42)
wsv069 (IE1) promoter	LvRelish	*L. vannamei*	(12)
wsv069 (IE1) promoter	LvHMGB, LvDorsal	*L. vannamei*	(208)
wsv069 (IE1) promoter	LvSpz4 mediated NF-κB activation	*L. vannamei*	(38)
wsv069 (IE1), wsv303, and wsv371 promoters	LvMyD88	*L. vannamei*	(44)
wsv069 (IE1), wsv303, and wsv371 promoters	LvPellino	*L. vannamei*	(57)
wsv069 (IE1), wsv303, and wsv371 promoters	LvTube, wsv390	*L. vannamei*	(47)
wsv069 (IE1) and wsv303 promoters	LvRelish, LvDorsal	*L. vannamei*	(53)
wsv069 and wsv249 promoters	Lvc-Jun, Lvc-Fos	*L. vannamei*	(119)
wsv051, wsv056, wsv069, wsv078, wsv079, wsv080, wsv083, wsv091, wsv094, wsv098, wsv100, wsv101, wsv103, wsv108, wsv178, wsv249, wsv358, wsv403, and wsv465 promoters	LvDorsal	*L. vannamei*	(209)
wsv051, wsv059, wsv069, wsv083, wsv090, wsv107, wsv244, wsv303, wsv371, and wsv445 promoters	LvIKKβ, LvIKKε	*L. vannamei*	(52)
wsv056, wsv069, wsv078, wsv079, wsv080, wsv083, wsv091, wsv094, wsv098, wsv099, wsv101, wsv103, wsv108, wsv178, wsv187, wsv249, wsv358, wsv403, and wsv465 promoters	LvMKK6	*L. vannamei*	(123)

Viruses have a very limited set of genes and, therefore, must use the host cellular resources to achieve their life cycles. WSSVs have evolved to use the activation of host NF-κB pathways—more specifically via the transcription factors Dorsal, Relish, c-Jun, and/or c-Jun—to promote its self-gene expression and genome replication. However, the activation of host NF-κB pathways leads to boost the synthesis of a specific set of AMPs or effectors with antiviral activities against WSSV. This is not inconceivable, but it seems apparent that WSSVs have evolved some unknown strategies to attenuate the antiviral role of host NF-κB pathways, but instead engage its activation to favor viral pathogenesis. This could be explained by the observation that many NF-κB-controlled AMPs are induced in the stage of early infection but are quickly inhibited later. However, the function of NF-κB pathways during other viral infection is still poorly understood. PmRelish from *P. monodon* seems to play an antiviral role against Yellow head virus (YHV) (114). More studies regarding the roles of host-signaling pathways in WSSV and other viral infections should be performed.

## Conclusions and Future Studies

The innate immune system is of great importance for shrimp to defend against infection. Recently, studies on two NF-κB pathways in shrimp mainly contain the follow themes: the identification and characterization of components of NF-κB pathways; dissecting signal transduction components of NF-κB pathways; and determining the function of NF-κB pathways in response to bacterial and viral (WSSV) infection. However, there are also many key components that have not been uncovered, especially in the IMD pathway, such as the receptors for DREDD and FADD, and how the action of signal transduction in this pathway is mediated is still unclear. Recently, the genome sequence of *L. vannamei*, with the annotation of 25,596 protein-coding genes, has been reported (214). Decoding the *L. vannamei* genome will not only promote the discovery of some conserved components of signaling pathways, but will also provide the opportunity to understand various biological processes of shrimp at the genomic level. In the *Drosophila* genome, a total of nine Tolls have been found, but only the Toll1, or simply Toll, has been definitively identified as an upstream receptor of Dorsal or Dif. Until now, there have been five groups of Tolls identified in shrimp, but which of these Tolls can induce Dorsal or other transcription factors, such as ATF and IRF activation, is still unclear. Additionally, different from how *Drosophila* Toll recognizes infection via binding to Spätzle, shrimp Tolls can directly sense foreign pathogenic motifs similar to that of mammals. For example, three Tolls from *M. japonicas* can directly bind to both PGN and LPS (73) and two Tolls from *L. vannamei* can interact with CpG ODN 2395 (72) *in vitro*. Therefore, understanding how shrimp Toll receptors recognize invading pathogens will provide novel insights into the sensing of Tolls in invertebrates.

Additionally, most of these studies have only reported the phonotype of functions, which are insufficient to present the undergoing mechanisms of these genes mediated immune response. In mammals, crosslinking of different signaling pathways, such as NF-κB related pathways and IRF-related pathways, make the innate immune system a complex network for coordinating appropriate immune responses. Studies on the interplay between Toll and IMD pathways, as well as the interplay among other pathways, should be conducted in shrimp in future studies. Furthermore, it is generally accepted that most AMPs in shrimp could be regulated by both Toll and IMD pathways, but research in this field is still in its infancy and additional experiments are required to explore the individual roles of the two pathways in regulating AMPs. As mentioned above, the strategies by which WSSVs attenuate the antiviral role of host NF-κB pathways to instead engage its activation in favor of viral pathogenesis is still poorly characterized. Further studies on the relationship between host and viral proteins need to be investigated in detail, which will help us to better understand viral pathogenesis and to develop effective strategies for viral-disease control.

## Author Contributions

All authors listed have made a substantial, direct and intellectual contribution to the work, and approved it for publication.

### Conflict of Interest Statement

The authors declare that the research was conducted in the absence of any commercial or financial relationships that could be construed as a potential conflict of interest.
